# A Novel Four-Gene Prognostic Signature for Prediction of Survival in Patients with Soft Tissue Sarcoma

**DOI:** 10.3390/cancers13225837

**Published:** 2021-11-21

**Authors:** Changwu Wu, Siming Gong, Georg Osterhoff, Nikolas Schopow

**Affiliations:** 1 Institute of Anatomy, University of Leipzig, Liebigstraße 20, 04103 Leipzig, Germany; changwu.wu@studserv.uni-leipzig.de (C.W.); nikolas.schopow@medizin.uni-leipzig.de (N.S.); 2 Sarcoma Center, Department of Orthopedics, Trauma and Plastic Surgery, University Hospital Leipzig, 04103 Leipzig, Germany; georg.osterhoff@medizin.uni-leipzig.de

**Keywords:** soft tissue sarcomas, gene signature, prognosis, nomogram

## Abstract

**Simple Summary:**

Soft tissue sarcomas (STS) still lack effective clinical stratification and prognostic models. The aim of this study is to establish a reliable prognostic gene signature in STS. Using 189 STS samples from the TCGA database, a four-gene signature (including DHRS3, JRK, TARDBP and TTC3) and nomograms that can be used to predict the overall survival and relapse free survival of STS patients was developed. The predictive ability for metastasis free survival was externally verified in the GEO cohort. We demonstrated that the novel gene signature provides an attractive platform for risk stratification and prognosis prediction of STS patients, which is of great importance for individualized clinical treatment and long-term management of patients with this rare and severe disease.

**Abstract:**

Soft tissue sarcomas (STS), a group of rare malignant tumours with high tissue heterogeneity, still lack effective clinical stratification and prognostic models. Therefore, we conducted this study to establish a reliable prognostic gene signature. Using 189 STS patients’ data from The Cancer Genome Atlas database, a four-gene signature including DHRS3, JRK, TARDBP and TTC3 was established. A risk score based on this gene signature was able to divide STS patients into a low-risk and a high-risk group. The latter had significantly worse overall survival (OS) and relapse free survival (RFS), and Cox regression analyses showed that the risk score is an independent prognostic factor. Nomograms containing the four-gene signature have also been established and have been verified through calibration curves. In addition, the predictive ability of this four-gene signature for STS metastasis free survival was verified in an independent cohort (309 STS patients from the Gene Expression Omnibus database). Finally, Gene Set Enrichment Analysis indicated that the four-gene signature may be related to some pathways associated with tumorigenesis, growth, and metastasis. In conclusion, our study establishes a novel four-gene signature and clinically feasible nomograms to predict the OS and RFS. This can help personalized treatment decisions, long-term patient management, and possible future development of targeted therapy.

## 1. Introduction

Soft tissue sarcomas (STS) are a group of rare malignant tumours mainly derived from the embryonic mesoderm, with high tissue heterogeneity in each subtype, that represent 2% of all adult, and 7% of all childhood cancers [1,2]. At present, more than 100 tumour subtypes of STS have been identified, the most common of which are liposarcoma, including dedifferentiated liposarcoma (DDLPS), leiomyosarcoma (LMS) and undifferentiated polymorphic sarcoma (UPS) [3,4]. The prognosis of STS patients remains poor. The five-year overall survival rate for advanced STS is less than 20% [5]. Surgical resection with radiotherapy is the most effective treatment strategy for early localized STS, and chemotherapy is usually indicated for patients with metastatic tumours [6,7,8]. Previous studies have shown that risk stratification and targeted therapy can significantly improve the treatment effect for most tumours, and STS is no exception [9,10]. Accurately stratifying the risk of STS patients is helpful for long-term personalized management of patients and selection of appropriate treatment strategies [7,10]. The previous view was that histological grading was the most important prognostic stratification indicator for STS [11]. However, considering the huge heterogeneity, extremely low incidence and complex biological behaviour of STS, grade is not always a reliable parameter; more efficient and robust prognostic prediction models are required. Molecular biomarkers play a key role in prognosis and treatment decisions for a variety of tumours; for example, PD-1 and PD-L1 are not only prognostic markers for a variety of tumours, but also key indicators for immune checkpoint therapy [12]. In STS, molecular signatures, including hypoxia-related gene signatures, have also been shown to play a role in risk classification [13]. The identification of new molecular signatures and their combination with existing predictors is expected to improve the identification of “high-risk” patients [14].

With the development of gene sequencing technology, many analyses in public databases have shown that gene markers at the messenger RNA (mRNA) level have great potential in the risk stratification and prognosis prediction of various tumours. For example, Zuo et al. established a six-gene signature to predict both disease-free and overall survival in patients with non-small cell lung cancer and Xiao et al. developed a three-gene signature as a prognostic biomarker for low-grade glioma [15,16]. Due to the extremely low incidence of STS, it is hard for a single institution to collect enough patient tumour tissue and clinical information to establish a prognostic gene signature. At this time, in-depth mining of publicly available gene sequencing data is often an effective way to establish a robust prognostic gene signature.

For this study, data from The Cancer Genome Atlas (TCGA) database was used to create a gene signature of STS to predict survival and outcome. In addition, data from the Gene Expression Omnibus (GEO) was used as an independent cohort to perform an external validation. We demonstrated that the novel gene signature provides an attractive platform for risk stratification and prognosis prediction of STS patients, which is of great importance for individualized clinical treatment and long-term management of patients with this rare and deadly disease.

## 2. Materials and Methods

### 2.1. Patient Data

The RNA-seq (IlluminaHiSeq 2000) data of 265 STS patients was obtained from TCGA through the University of California, Santa Cruz, Xena Functional Genomics Explorer (https://xenabrowser.net/datapages/ (accessed on 5 January 2021)). The RNA-seq data are level 3 data from the TCGA data coordination centre, and the RNA-Seq by Expectation Maximization normalized count converted by log2(x + 1) shows the transcription estimate at the gene level. The latest clinical pathology and survival data were obtained from a publication of the TCGA Network [17]. Due to the difficulty in extracting sufficient RNA, well-differentiated liposarcoma was not included in this study [17]. After filtering the data, only samples that had complete follow-up and clinical information, including age at first diagnosis, gender, pathological tumour size, whether radiotherapy and chemotherapy were performed, FNCLCC grade, vital status, OS time, relapse status, RFS time and histological type were included. The patients had no history of systemic chemotherapy or radiotherapy before tumour resection. The gene expression profile and MFS data of 309 STS samples from the GSE21050 dataset were obtained through the GEO database (https://www.ncbi.nlm.nih.gov/geo/ (accessed on 26 January 2021)) to further verify the prognostic ability of the gene signature [18], in which the gene expression data was converted by log2(x + 1) for normalization. The difference of DHRS3, JRK, TARDBP and TTC3 expression between STS tissues and normal tissues was analyzed in the Oncomine database (https://www.oncomine.org/resource/main.html (accessed on 21 April 2021)).

### 2.2. Establishment of Prognostic Gene Signature

For this study patients were divided into a training set (*n* = 95) and a testing set (*n* = 94) by random assignment. In the training set, a robust likelihood-based survival analysis was performed using the package “rbsurv” in the R software (Version 4.0.3, R Foundation for Statistical Computing, Vienna, Austria) to identify genes related to OS [19]. After 10 iterations, 22 prognostic-related genes were obtained. Then, the R packages “glmnet” and “survival” were used to perform LASSO model analysis and multivariate Cox regression analysis and determined a survival-related prognostic model under the threshold of *p* < 0.05. In addition, the “survminer” and “survival” R packages were used to find the best cut-off value to classify samples into high and low risk groups. The Kaplan–Meier curve was used to estimate the difference in OS and RFS between the two groups and used the R package “timeROC” to plot the receiver operating characteristic (ROC) curve over time to assess the predictive value of this prognostic gene signature. Finally, the predictive value of prognostic gene signatures was verified in the testing set and the whole set.

### 2.3. Establishment of Predictive Nomograms

In order to establish easy-to-use and clinically adaptable prognostic prediction models, we used multivariate Cox regression analysis to identify independent risk factors related to the prognosis of STS and combined these independent prognostic factors using the R package “rms” to construct predictive nomograms. Then, calibration curves were plotted to evaluate the deviation between the estimated OS/RFS probability and the actual OS/RFS probability, and the C-index was used to evaluate the prognostic accuracy of the nomograms.

### 2.4. Gene Set Enrichment Analysis 

To explore the potential molecular mechanism of the prognostic gene signature, GSEA software (Version 4.1.0) was used to perform gene set enrichment analysis by using MSigDB C2 CP: Canonical pathways gene set collection [20]. The significance threshold was set to *p* < 0.05, false discovery rate (FDR) < 0.25.

### 2.5. Statistical Analysis

Univariate and multivariate Cox regression were performed to evaluate the impact of various potential risk factors on OS and RFS. A Kaplan–Meier curve was used for data stratification analysis and a log-rank test was used to evaluate its statistical significance. The R packages “cowplot” and “pheatmap” were used to generate risk distribution plots. All statistical calculations were performed using R software, *p* < 0.05 was considered statistically significant.

## 3. Results

### 3.1. Establishment of Four-Gene Prognostic Signature

**Figure 1:**
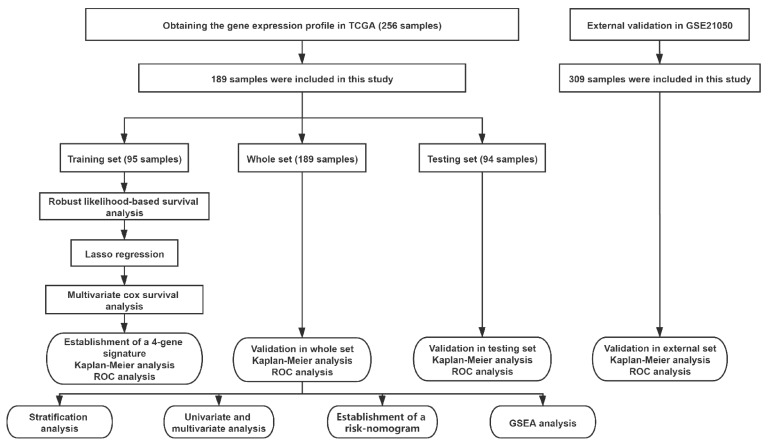
Workflow of the analyses.

**Figure 2:**
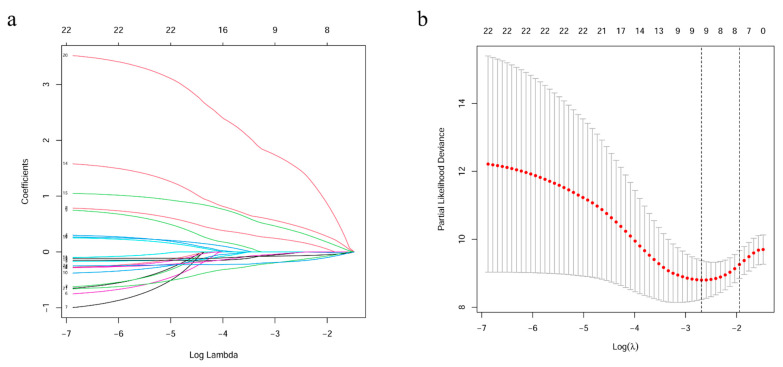
Identification of 22 survival-related genes by LASSO regression. (**a**) LASSO coefficient profiles of 22 prognostic genes. (**b**) Ten-time cross-validation for tuning parameter selection in the LASSO model.

A flow chart of this study is presented in Figure 1. Data on 256 sarcomas are published in TCGA, of which 206 sarcomas are well characterized fully revised. Of these, tumour size is missing for eight patients and chemotherapy and radiation information is missing for nine additional patients. In total, 189 patients were included for final analysis (Supplementary Table S1). The patients had an average age of 60 years at diagnosis and 95% of them suffered from a high-grade STS (Table 1). The patients had no history of systemic chemotherapy or radiotherapy before tumour resection; this means that the gene expression cannot have been falsified in this way [21]. First, a robust likelihood-based survival analysis was performed on 95 samples in the training set and obtained 22 genes that are significantly related to overall survival (OS). Subsequently, LASSO Cox regression analysis was performed, further narrowing down the number of these survival-related genes to nine genes (Figure 2). To further narrow the scope of mRNA, a multivariate Cox proportional hazard regression analysis was performed. In the end, four genes were identified and used them for the construction of prognostic gene signature. The four genes identified in this context were dehydrogenase/reductase 3 (DHRS3), Jrk helix-turn-helix protein (JRK), TAR DNA binding protein (TARDBP) and tetratricopeptide repeat domain 3 (TTC3). Then, the relationship between the expression levels of these four genes and OS was explored, and it was found that patients with high expression of JRK, TARDBP and TTC3 had shorter OS, while patients with high expression of DHRS3 had better OS (Supplementary Figure S1). In addition, gene expression analysis based on the Oncomine database showed that JRK, TARDBP and TTC3 expression in STS tissues were higher than normal tissues, while DHRS3 was the opposite (Supplementary Figure S2). This implies the role of these four genes in the occurrence and development of STS. According to the multivariate Cox regression model, the correlation coefficients of the four genes (expression level) were estimated and the four-gene signature (4GS)-risk score of each patient was also calculated.

4GS-risk score = −0.3059 × [DHRS3] + 0.8152 × [JRK] + 3.4023 × [TARDBP] + 1.0905 × [TTC3]. (1)

### 3.2. The Predictive Value of Four-Gene Signature on the OS of STS Patients

**Figure 3:**
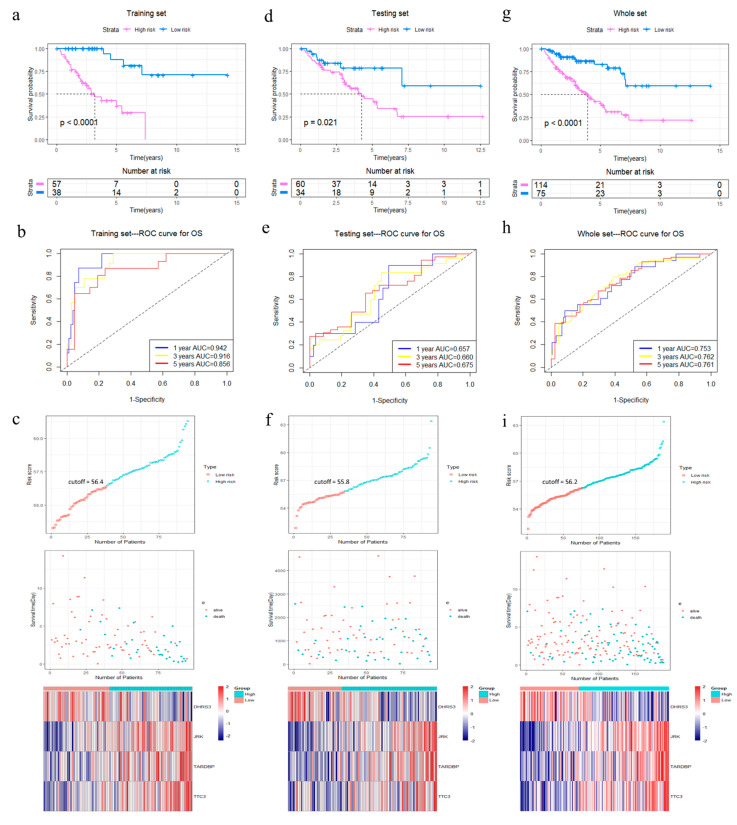
Analysis of OS for the four-gene signature in STS. (**a**–**c**) Kaplan–Meier curve, time-dependent ROC analysis, risk score, survival status and heatmap of mRNA expression of the four-gene signature in the training set. (**d**–**f**) Kaplan–Meier curve, time-dependent ROC analysis, risk score, survival status and heatmap of mRNA expression of the four-gene signature in the testing set. (**g**–**i**) Kaplan–Meier curve, time-dependent ROC analysis, risk score, survival status and heatmap of mRNA expression of the four-gene signature in the whole set of TCGA cohort. Significance for survival analysis was calculated using a log-rank test, with the red line representing the 4GS-high-risk group and the blue line representing the low-risk group. Red in the heatmap represents high expression and blue represents low expression.

**Figure 4:**
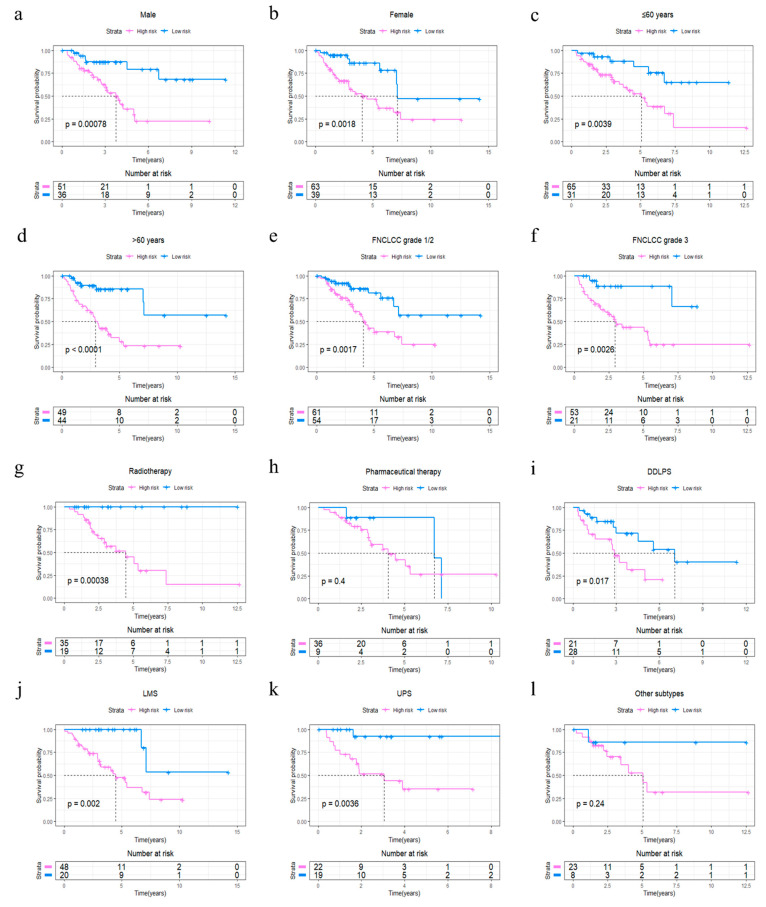
Stratified OS analysis based on the risk model in the whole set of TCGA cohort. Based on the risk score model, stratified OS analysis performed in patients with different clinical parameters, such as gender (**a**,**b**), age group (**c**,**d**), FNCLCC grade (**e**,**f**), radiotherapy (**g**), pharmaceutical therapy (**h**) and histological type (**i**–**l**) in the whole set of TCGA cohort. Significance for survival analysis was calculated using a log-rank test, with the red line representing the 4GS-high-risk group and the blue line representing the low-risk group. The grouping of STS samples is shown at the bottom of the charts.

The R package “survminer” and “survival” were used to find the best cut-off value for the risk score to divide patients into 4GS-high-risk and 4GS-low-risk groups, and we performed the same operations on the testing set and the whole set. In the training set, the Kaplan–Meier curve showed that the OS of the 4GS-high-risk group was significantly shorter than that of the low-risk group (*p* < 0.0001) (Figure 3a). In addition, the ROC curve showed that the area under the curves (AUC) for 1-, 3- and 5-year OS were 0.942, 0.916, and 0.856, respectively (Figure 3b). In Figure 3c, we analysed the risk score distribution, survival status distribution and four-genes expression distribution of patients in the training set. The heatmap showed that the expression of DHRS3 was lower in the high-risk group, while the expression of JRK, TARDBP and TTC3 were obviously higher. We obtained similar results in the testing set and the whole set. In the testing set and the whole set, the high-risk groups had both shorter OS (*p* = 0.021 & *p* < 0.0001) (Figure 3d,g). The AUCs of 1-, 3-, and 5-year OS were 0.657, 0.660, 0.675 in the testing set, and 0.753, 0.762, 0.761 in the whole set (Figure 3e,h). The risk score distribution, survival status distribution and four genes expression distribution of patients are shown in Figure 3f,i, respectively. For the whole set, a data stratification analysis was also conducted. These patients were stratified according to different clinical parameters, such as gender, age (≤60 years/>60 years), Fédération Nationale des Centres de Lutte Contre le Cancer (FNCLCC) grade (grade 1&2/grade 3), radiotherapy, pharmaceutical therapy, histological type (DDLPS/LMS/UPS/other subtypes), tumour size (≤10 cm/>10 cm) and site of tumour. The results of stratified analysis showed that in patients of different age groups, gender, FNCLCC grade groups, and tumour size groups, the 4GS-high-risk groups all had lower OS (all *p* < 0.05) (Figure 4a–f and Supplementary Figure S3a,b). For patients who had undergone radiotherapy, the 4GS-high-risk group also had significantly shorter OS (*p* = 0.00038) (Figure 4g). Among patients who had undergone pharmaceutical therapy, there was no statistical difference between 4GS-high-risk patients and low-risk patients due to the small sample size of the low-risk group (Figure 3h). For patients with DDLPS, LMS and UPS, 4GS-high-risk patients also all had shorter OS than low-risk patients (all *p* < 0.05) (Figure 4i–k). In other subtypes, there was no statistical difference due to the small sample size of 4GS-low-risk patients (Figure 4l). Additionally, for patients with a tumour in retroperitoneum, upper/lower extremity and other site, 4GS-high-risk patients had shorter OS (all *p* < 0.05) (Supplementary Figure S3c,d,f). Patients with a tumour in superficial trunk, 4GS-high-risk patients also had shorter OS but no statistical difference due to the sample size limitation (Supplementary Figure S3e). Overall, our results indicated that the four-gene signature performed well in OS prediction.

### 3.3. The Predictive Value of Four-Gene Signature on the Relapse Free Survival (RFS) and Metastasis Free Survival (MFS) of STS Patients

**Figure 5:**
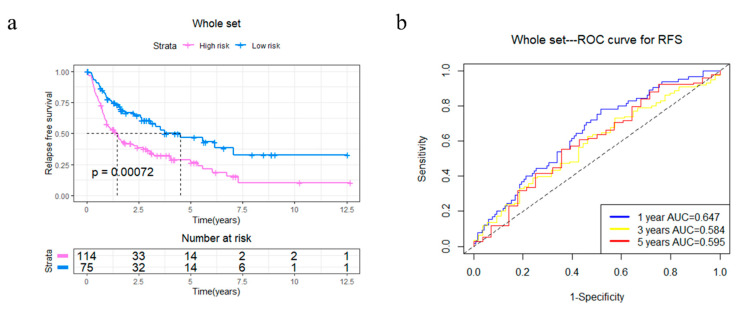
Kaplan–Meier analysis and time-dependent ROC analysis of RFS for the four-gene signature in STS. (**a**) Kaplan–Meier curve in the whole set. The significance was calculated using a log-rank test, with the red line representing the high-risk group and the blue line representing the low-risk group. The grouping of STS samples is shown at the bottom of the charts. (**b**) Time-dependent ROC curve in the whole set (AUC: 0.647, 0.584, and 0.595; 1, 2, and 5 years RFS).

In order to explore the potential capabilities of the four-gene signature in RFS prediction, 4GS-high-risk and low-risk patients were compared in the whole TCGA set. The Kaplan–Meier curve showed that 4GS-high-risk patients had significantly shorter RFS, suggesting that these patients had a higher recurrence rate (*p* = 0.00072) (Figure 5a). Subsequently, the ROC curve showed that the AUCs of 1-, 3- and 5-year RFS were 0.647, 0.584 and 0.595, respectively (Figure 5b). Based on different clinicopathological characteristics, data stratification was also carried out for further analyses. The results showed that 4GS-high-risk patients had significantly shorter RFS in the male and female group, >60 years group, FNCLCC grade 3 group, >10 cm tumour size group, and upper/lower extremity group (all *p* < 0.05) (Supplementary Figures S4a,b,d,f and S5a,d). Although there was no statistical significance in the ≤60 years group, FNCLCC grade 1/2 group, ≤10 cm tumour size group, retroperitoneum, superficial trunk and other site groups, the four-gene signature still showed a tendency to indicate different prognoses (Supplementary Figures S4c,e and S5b,c,e,f). For patients who had undergone radiotherapy, the 4GS-high-risk group had significantly shorter RFS (*p* = 0.0055) (Supplementary Figure S4g). Similar to predicting OS, due to the small sample size of 4GS-low-risk patients, there was only a trend that high-risk group had lower RFS in the chemotherapy group and other subtype groups without statistical significance (Supplementary Figure S4h,l). In addition, there was only a tendency for DDLPS patients, which suggested that expanding the sample size may better confirm the predictive value for RFS (Supplementary Figure S4i). For LMS and UPS patients, the high-risk groups also had significantly shorter RFS (all *p* < 0.05) (Supplementary Figure S4j,k). Collectively, our results showed that the four-gene signature had a good effect even in predicting RFS. To further verify the effectiveness of the prognostic model, as an independent dataset containing complete OS and RFS data could not be found, the GSE21050 dataset from the GEO database was used to validate the prediction efficacy of the four-gene signature in MFS [14]. The Kaplan–Meier curve showed that 4GS-high-risk patients have significantly worse MFS than 4GS-low-risk patients (*p* = 0.00081) (Supplementary Figure S6), while the ROC curve showed that the 1-, 3-, and 5-year MFS AUCs were 0.593, 0.615, and 0.621, respectively (Supplementary Figure S6). This result implies that in an independent dataset, the four-gene signatures also have good prognostic prediction capabilities.

### 3.4. Univariate and Multivariate Analyses of the Four-Gene Signature Prognostic Role 

In order to verify the independent prognostic effect of the four-gene signature, Cox univariate and multivariate analyses were performed. Age, gender, pathological tumour size, radiotherapy, pharmaceutical therapy, FNCLCC grade, site of tumour, histological subtype and the risk score calculated based on the four-gene signature were used as the covariates for the analysis. Finally, univariate, and multivariate analyses indicated that age, pathological tumour size and 4GS-risk score were independent prognostic factors for OS in STS patients (Table 2). In addition, pathological tumour size, pharmaceutical therapy, FNCLCC grade, and 4GS-risk score were independent prognostic factors for RFS in STS patients (Table 3).

### 3.5. Establishment of Nomograms for Predicting OS and RFS in STS Patients

**Figure 6:**
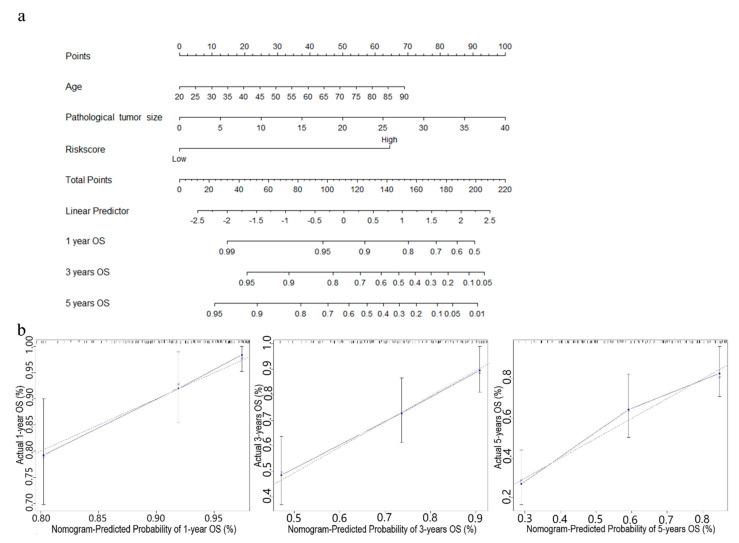
Nomogram predicting OS for STS patients. (**a**) The nomogram for predicting probabilities of patients with 1-, 3-, and 5-year OS. (**b**) The calibration plot for internal validation of the nomogram. The Y-axis represents actual survival, and the X-axis represents nomogram-predicted survival. The dashed diagonal line represents the ideal nomogram.

**Figure 7:**
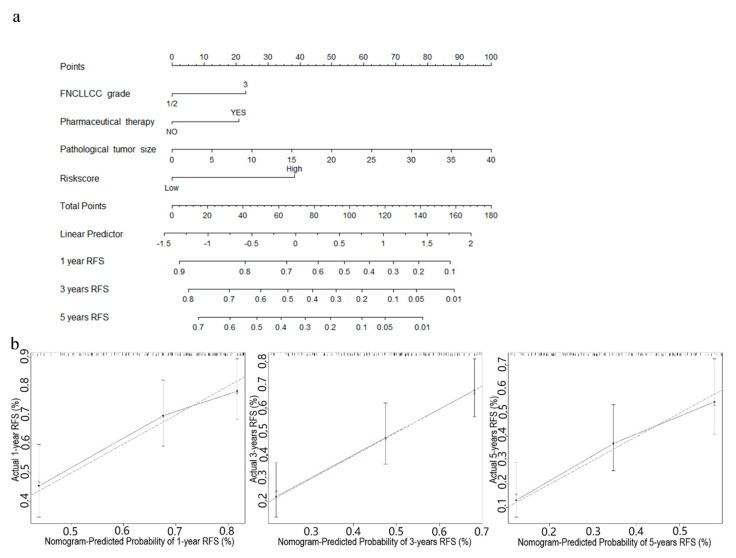
Nomogram predicting RFS for STS patients. (**a**) The nomogram for predicting probabilities of patients with 1-, 3-, and 5-year RFS. (**b**) The calibration plot for internal validation of the nomogram. The Y-axis represents actual survival, and the X-axis represents nomogram-predicted survival. The dashed diagonal line represents the ideal nomogram.

In order to further improve the prediction accuracy of the four-gene signature, other independent prognostic factors were combined in multivariate analyses to establish clinically adaptable prognostic nomograms for OS and RFS. As shown in Figure 6a and Figure 7a, the higher the total score based on the sum of the assigned numbers of each factor in the nomograms, the worse the 1-, 3- and 5-year OS rates and RFS rates. Subsequently, the nomograms were evaluated through the C-statistic discriminant indexes and the calibration plots. The C-index values used to evaluate the OS model and RFS model were 0.744 and 0.660, respectively. The higher C-index values indicated the robustness of the nomograms. The calibration curves showed that the predicted values and the actual values had a very satisfactory agreement in the probability of 1-, 3-, 5-year OS and RFS (Figure 6b and Figure 7b). This fully illustrated the potential value of predicting nomograms in clinical guidance.

### 3.6. Identification of Four-Gene Signature Related Biological Pathways and Processes

**Figure 8:**
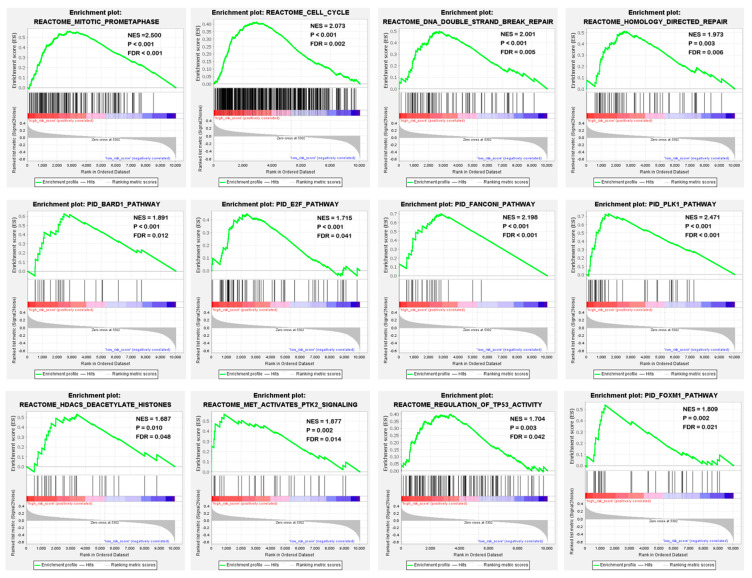
Four-gene signature associated biological processes and signal transduction pathways based on the GSEA of the TCGA cohort. Red represents the high-risk group and blue represents the low-risk group.

To identify potential biological processes and signal transduction pathways, GSEA based on the risk scores calculated by the four-gene signature was performed. The specific results were shown in Supplementary Table S2. In high-risk patients, enrichment of cancer-related biological processes was found, including mitosis, cell cycle, DNA double-strand damage repair and homologous repair. In addition, they showed an activation of key pathways in tumorigenesis, development, and metastasis, such as BARD pathway, E2F pathway, Fanconi pathway, TP53 pathway and PLK1 pathway (Figure 8). This suggested that the four-gene signature may indeed be involved in the progression of sarcoma.

## 4. Discussion

Precisely predicting of the disease progression is one of the main challenges of STS. Considering the heterogeneity of STS, there is an urgent need to identify new prognostic biomarkers and establish more practical prognostic models. Recently, gene prognostic signatures based on aberrant mRNA have received widespread attention and have shown great potential in non-small cell lung cancer, pancreatic ductal adenocarcinoma, hepatocellular carcinoma and other malignant tumours [15,16,22,23]. With this study, a prognostic gene signature for STS could be identified using a systematic analysis of RNA-seq data in the TCGA database and externally validated on an independent cohort.

We determined a four-gene prognostic signature including DHRS3, JRK, TARDBP and TTC3 by consecutively examining robust likelihood-based survival analysis, LASSO Cox regression analysis and multivariate Cox proportional hazard regression analysis and determined its prognostic value by Kaplan–Meier curves, ROC curves and data stratification analyses. This four-gene signature has excellent prognostic performance in several major pathological subtypes including DDLPS, LMS and UPS. Among them, because LMS can be further divided into soft tissue leiomyosarcoma (STLMS) and uterine leiomyosarcoma (ULMS), OS/RFS analysis was performed on these two subtypes, respectively. In STLMS, the OS/RFS of 4GS-high-risk patients was also worse than that of 4GS-low-risk patients (Supplementary Figure S7a), while in ULMS, due to the sample size limitation of low-risk patients, there is no statistical difference (Supplementary Figure S7b). In addition, Shen et al. recently reported a 19-gene prognostic signature for STS [24]. Compared with this gene signature, the ROC curve showed that our four-gene signature had better performance in both short-term and long-term predictions (Supplementary Figure S8).

In our four-gene signature, TARDBP was found to have the largest positive coefficient value, which indicated that TARDBP may play an important role in STS. TARDBP is known to be an RNA-binding protein related to neurodegenerative diseases such as frontal temporal lobe degeneration and amyotrophic lateral sclerosis and may play an important role in cell metabolism including glucose metabolism and lipid metabolism [25,26,27,28,29]. Recent studies have shown that TARDBP plays an important role in many tumours, including leukaemia, Ewing sarcoma and hepatocellular carcinoma (HCC) [30,31,32,33]. It regulated glycolysis of cancer cells through transcriptional inhibition, resulting in a poor prognosis of HCC [33]. However, the role and mechanism of TARDBP in STS have not been reported yet, so future studies are urgently needed. In addition to TARDBP, JRK and TTC3 also had higher positive coefficient values. JRK is a homolog of the Earthbound 1 [34]. It has been found to have abnormally elevated expression in colorectal, breast and ovarian cancer, and is related to the increased expression of β-catenin target genes and increased cell proliferation [35]. It is considered a potential new oncogene and cancer treatment target [35]. However, the role of JRK in STS remains unclear. TTC3, a ubiquitin E3 ligase, was found to promote the degradation of ubiquitination and phosphorylation of Akt, which is related to the clinical symptoms of Down syndrome [36]. A recent study found that TTC3 may contribute to the epithelial-mesenchymal transition and myofibroblast differentiation induced by transforming growth factor-β [37]. Dey-Guha et al. found that TTC3-mediated proteasome-dependent degradation was involved in the β1 integrin/FAK/mTORC2/AKT1-related signalling pathway, thereby mediating the chemo-resistance of breast cancer cells [38]. The chemotherapy response rate in TCGA is not documented, therefore we could only suspect and not prove a similar association between TTC3 and a worse chemotherapy response rate in STS. DHRS3 is the only gene with a negative coefficient value in the four gene signatures. As a highly conservative member of the short chain alcohol dehydrogenase/reductase superfamily, it is involved in the metabolism of retinol. Retinol-like compounds mainly affect intracellular processes by regulating gene expression, including cell proliferation and differentiation [39]. Although DHRS3 was found to be upregulated in papillary thyroid cancer and neuroblasts, DHRS3 was negatively correlated with the metastasis of papillary thyroid carcinoma and was associated with better prognosis of neuroblastoma [40,41]. This is consistent with the negative coefficient of DHRS3 in our study, but to understand the specific role of DHRS3 in STS further research is necessary. Notably, these four genes were identified based on real patient cohorts, and thus may be more feasible for clinical application and should be validated prospectively, which is important for further clinical translation and evaluation.

In this study, through univariate and multivariate analysis, it was determined that the risk score calculated based on the four-gene signature was an independent prognostic factor for OS and RFS in STS patients. In addition, age and pathological tumour size have also been determined to be independent prognostic factors for OS in STS patients, while pathological tumour size, pharmaceutical therapy, and FNCLCC grade were independent prognostic factors for RFS. Notably, pharmaceutical therapy was a poor prognostic factor in patients with STS in terms of RFS (Table 3). In fact, there has been a lot of evidence proving that chemotherapy may be a sharp double-edged sword. The underlying mechanism may be the resistant selection and disseminating cancer stem cells induced by chemotherapy at the site of the primary tumour, chemotherapy-induced recruitment of immune cells conducive to the dissemination of primary tumour cells, and mobilization/regulation of circulating tumour cell heterogeneity [42,43,44,45]. In terms of STS, in a recent study, researchers found that adjuvant chemotherapy was a poor prognostic factor for disease-free survival in chest wall STS patients (HR: 2.797; 95% CI: 1.057–3.409; *p* = 0.04), which was consistent with our results [46]. Meta-analyses also cannot prove the advantage of cytotoxic treatment in STS. In 3157 patients with localized STS, no advantage of resection and chemotherapy over surgery alone was demonstrated [43]. In advanced STS, Zer et al. showed at least a weakly significant advantage of combined doxorubicin-based over doxorubicin alone chemotherapy in 5044 patients [47]. In contrast, Tanaka et al. demonstrated only an increase in side effects but no significant overall survival benefit in 6156 patients [44]. The use of chemotherapy in patients with STS should always be decided on a case-by-case basis, and scientific selection of chemotherapeutic drugs and appropriate dose selection are particularly important. This decision could be supported by the four-gene signature.

Nomograms are widely used to predict the prognosis of cancer [48]. Previous studies have shown some nomograms about STS [49]. For a single patient, multiple nomograms can be used to calculate the prognosis [49]. However, there is currently no nomogram combined with gene signature available in STS. After combining the 4GS-risk score with other independent prognostic factors, clinically applicable nomograms were established for both OS and RFS. The calibration plots confirmed that predictive nomograms had good accuracy and stability in predicting OS and RFS. In addition, GSEA revealed some obviously enriched intracellular processes and signal pathways. Many studies have shown that cell cycle, DNA double-strand damage repair, E2F pathway, TP53 pathway and PLK1 pathway, etc. play important roles in tumour progression [50,51,52,53]. These GSEA-enriched important tumour-related signal pathways supported the hypothesis that the four-gene signature does have OS and RFS predictive capabilities and confirmed the reliability and rationality of the gene signature. Mechanistically, the four-gene signature may influence tumour progression through oncogenic-related pathways such as the E2F, TP53 and PLK1 pathways, thereby affecting the prognosis of STS patients. In addition, it also provided potential methods for the development of possible targeted therapies in the future. 

As far as we know, the prognostic model and nomograms related to this four-gene signature have not been reported yet. This is a useful tool for risk stratification and prognosis prediction of STS patients and has potential significance for individualized treatment and long-term patient management. For example, it may make sense to increase the frequency of re-examination and adjust the chemotherapy regimen for patients who are predicted to have a high probability of recurrence. Based on previous studies [10], patients identified as high risk in this model require more intensive and longer-lasting chemotherapy, while less chemotherapy is more beneficial for patients with low risk. In addition, the 4GS-risk score is based on the mRNA expression of only four genes rather than DNA methylation or alternative splicing events, which can be achieved using simple quantitative reverse transcription PCR or immunohistochemistry to obtain approximate risk results, avoiding unnecessary genome-wide sequencing. Judging from the coefficients of the four genes, DHRS3 has a negative coefficient, and JRK, TARDBP and TTC3 have positive coefficients. Therefore, the lower the expression of DHRS3, the higher the expression of JRK, TARDBP, and TTC3, the greater the risk. It is more cost-effective, routine, and easy to apply in practice. However, our research still has certain limitations. First of all, due to the problem of sample size, some stratified analyses have shown trends and cannot reflect statistical differences. We only used the MFS data from the independent GEO cohort to validate the prognostic predictive ability. These would require a larger external sample size for further verification. More data sets will be available to enhance our results in the future. Secondly, the independent prognostic ability, biological function, and potential mechanism of these four genes in STS need to be further clarified by functional experiments. In addition, STS contains more than 100 subtypes, and only a few most common subtypes such as DDLPS, LMS and UPS were included in this study, while other subtypes including well-differentiated liposarcoma was not included, therefore we could not verify the predictive role of this gene signature for other tumour subtypes. Accordingly, we believe that this gene signature is currently most applicable to DDLPS, UPS and LMS. Finally, a large number of prospective cohorts are needed to verify the risk classification and prognosis prediction ability of the gene signature. Despite these shortcomings, our results still confirmed the important value of the four-gene signature in the OS and RFS prediction of STS. 

## 5. Conclusions

This study used clinical big data to establish a novel and easy-to-use four-gene mRNA signature and nomograms to predict the OS and RFS, which may contribute to clinical individualized treatment decisions based on risk stratification in clinical practice and possible future targeted therapy development. The repeatability of the 4GS-risk score was confirmed in an independent cohort of 309 samples. The 4GS-risk score could be an instrument to help in the clinical case-by-case decision in the future of whether chemotherapy would be beneficial for the patient or not and which treatment regimen should be chosen ideally.

## Figures and Tables

**Table 1:** Clinical characteristics of STS patients in the TCGA cohort.

Characteristics	All (*n* = 189)	Training Set (*n* = 95)	Testing Set (*n* = 94)
**Age group (Median)**			
≤60 years	96 (50.79%)	48 (50.53%)	48 (51.06%)
>60 years	93 (49.21%)	47 (49.47%)	46 (48.94%)
**Gender**			
Male	87 (46.03%)	45 (47.37%)	42 (44.68%)
Female	102 (53.97%)	50 (52.63%)	52 (55.32%)
**Pathologic tumour size**			
≤10.5 cm	95 (50.26%)	52 (54.74%)	43 (45.74%)
>10.5 cm	94 (49.74%)	43 (45.26%)	51 (54.26%)
**Radiotherapy**			
Yes	54 (28.57%)	34 (35.79%)	20 (21.28%)
No	135 (71.43%)	61 (64.21%)	74 (78.72%)
**Pharmaceutical therapy**			
Yes	45 (23.81%)	26 (27.37%)	19 (20.21%)
No	144 (76.19%)	69 (72.63%)	75 (79.79%)
**FNCLCC grade**			
1	10 (5.29%)	5 (5.26%)	5 (5.32%)
2	105 (55.56%)	49 (51.58%)	56 (59.57%)
3	74 (39.15%)	41 (43.16%)	33 (35.11%)
**Vital status**			
Alive	117 (61.90%)	62 (65.26%)	55 (58.51%)
Dead	72 (38.10%)	33 (34.74%)	39 (41.49%)
**Relapse status**			
Relapse	114 (60.32%)	61 (64.21%)	53 (56.38%)
Non-Relapse	75 (39.68%)	34 (35.79%)	41 (43.62%)
**Histological type**			
DDLPS	49 (25.93%)	23 (24.21%)	26 (27.66%)
LMS	68 (35.98%)	34 (35.79%)	34 (36.17%)
UPS	41 (21.69%)	24 (25.26%)	17 (18.09%)
MFS	17 (8.99%)	6 (6.32%)	11 (11.70%)
SS	10 (5.29%)	5 (5.26%)	5 (5.32%)
MPNST	4 (2.12%)	3 (3.16%)	1 (1.06%)
**Tumour site**			
Retroperitoneum	81 (42.8%)	39 (41.1%)	42 (44.7%)
Upper/Lower Extremity	57 (30.2%)	30 (31.6%)	27 (28.7%)
Superficial Trunk	9 (4.8%)	4 (4.2%)	5 (5.3%)
Chest	10 (5.3%)	4 (4.2%)	6 (6.4%)
Uterus	19 (10.1%)	11 (11.6%)	8 (8.5%)
Other	13 (6.8%)	7 (7.3%)	6 (6.4%)

DDLPS: dedifferentiated liposarcoma; LMS: leiomyosarcoma; UPS: undifferentiated pleomorphic sarcoma; MFS: myxofibrosarcoma; SS: synovial sarcoma; MPNST: malignant peripheral nerve sheath tumour.

**Table 2:** Univariate and multivariate analysis of the prognostic value of the four-gene signature in terms of OS in STS.

Characteristics	Univariate Analysis			Multivariate Analysis		
	HR	95%CI	*p*-Value	HR	95%CI	*p*-Value
**Age** (Continuous)	1.020	1.003~1.039	0.025	1.025	1.007~1.043	0.005
**Gender** (Male vs. Female)	1.015	0.637~1.619	0.949			
**Pathological tumour size** (cm)	1.043	1.016~1.070	0.002	1.065	1.037~1.094	<0.001
**Radiotherapy** (Yes vs. No)	0.758	0.444~1.293	0.309			
**Pharmaceutical therapy** (Yes vs. No)	1.291	0.769~2.167	0.335			
**FNCLCC grade** (3 vs. 1/2)	1.583	0.995~2.519	0.053			
**Tumour site**						
Upper/Lower extremity	(Reference)					
Retroperitoneum	1.100	0.630~1.917	0.738			
Superficial trunk	1.023	0.302~3.462	0.971			
Chest	1.418	0.532~3.782	0.485			
Uterus	1.436	0.652~3.161	0.369			
Other	0.507	0.118~2.171	0.360			
**Histological subtype**						
DDLPS	(Reference)					
LMS	0.669	0.379~1.183	0.167			
UPS	0.768	0.387~1.527	0.452			
MFS	0.829	0.354~1.945	0.668			
Other	0.649	0.224~1.886	0.428			
**4GS-Risk score ** (High vs. Low)	3.813	2.084~6.975	<0.001	5.141	2.756~9.589	<0.001

**Table 3:** Univariate and multivariate analysis of the prognostic value of the four-gene signature in terms of RFS in STS.

Characteristics	Univariate Analysis			Multivariate Analysis		
	HR	95%CI	*p*-Value	HR	95%CI	*p*-Value
**Age** (Continuous)	1.002	0.989~1.015	0.754			
**Gender** (Male vs. Female)	1.176	0.813~1.699	0.390			
**Pathological tumour size** (cm)	1.032	1.010~1.054	0.004	1.052	1.028~1.076	<0.001
**Radiotherapy** (Yes vs. No)	1.033	0.685~1.558	0.875			
**Pharmaceutical therapy ** (Yes vs. No)	1.723	1.146~2.591	0.009	1.528	1.001~2.332	0.049
**FNCLCC grade** (3 vs. 1/2)	1.465	1.009~2.126	0.045	1.592	1.089~2.328	0.016
**Tumour site**						
Upper/Lower extremity	(Reference)					
Retroperitoneum	0.896	0.585~1.375	0.618			
Superficial trunk	0.473	0.145~1.545	0.215			
Chest	0.761	0.321~1.809	0.537			
Uterus	1.260	0.667~2.380	0.369			
Other	0.653	0.256~1.666	0.476			
**Histological subtype**						
DDLPS	(Reference)					
LMS	0.869	0.553~1.367	0.545			
UPS	0.829	0.479~1.435	0.504			
MFS	0.847	0.417~1.723	0.648			
Other	0.595	0.249~1.421	0.243			
**4GS-Risk score** (High vs. Low)	1.978	1.322~2.959	<0.001	2.173	1.405~3.362	<0.001

## Data Availability

The results shown here are in whole based upon data generated by the TCGA Research Network: https://www.cancer.gov/tcga (accessed on 5 January 2021) and GEO database: https://www.ncbi.nlm.nih.gov/geo/ (accessed on 26 January 2021).

## Supplementary Materials

The following are available online at https://www.mdpi.com/article/10.3390/cancers13225837/s1, Figure S1: The relationship between the expression levels of these four genes and OS in the whole set of TCGA cohort, Figure S2: Differences in the expression of DHRS, JRK, TARDBP and TTC3 between STS tissues and normal tissues in Oncomine database, Figure S3: OS analysis of different tumor size groups and tumor site groups based on the risk model in the whole set of TCGA cohort, Figure S4: Stratified RFS analysis based on the risk model in the whole set of TCGA cohort, Figure S5: RFS analysis of different tumor size groups and tumor site groups based on the risk model in the whole set of TCGA cohort, Figure S6: Kaplan–Meier analysis and time-dependent ROC analysis of MFS for the four-gene signature in STS, Figure S7: Kaplan–Meier analysis of OS (a) and RFS (b) for the four-gene signature in STLMS and ULMS, Figure S8: Comparison of our four-genes signature and 19 genes signature of Shen et al., Table S1: Clinical characteristics of 189 STS patients, Table S2: Gene set enrichment analysis for risk score based on the four-gene signature.
