# Supplementary material for: A Novel Four-Gene Prognostic Signature for Prediction of Survival in Patients with Soft Tissue Sarcoma
Source: Cancers (Basel). 2021 Nov 21;13(22):5837. doi: 10.3390/cancers13225837 (PMC8616347; doi:10.3390/cancers13225837)
Supplement: Supplementary file 1 [file cancers-13-05837-s001.zip › cancers-1442615-supplementary/Supplementary figure s1-s8.pdf]

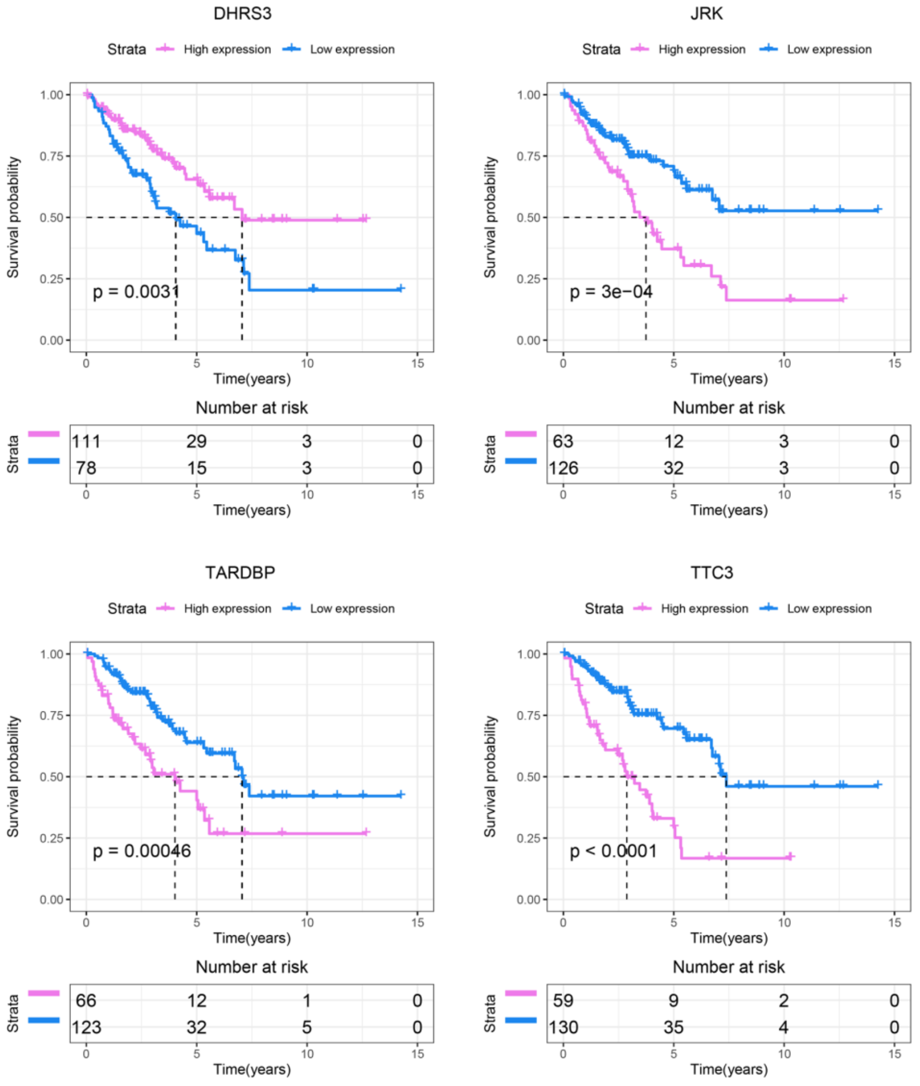

Supplementary Figure S1. The relationship between the expression levels of these four genes and OS in the whole set of TCGA cohort.

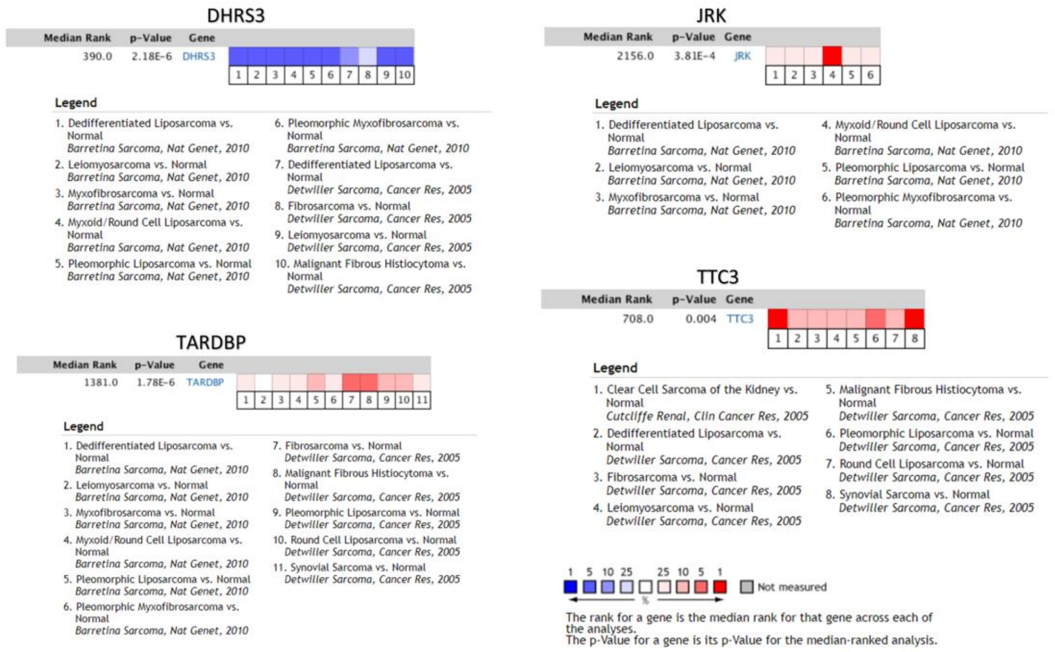

Supplementary Figure S2. Differences in the expression of DHRS, JRK, TARDBP and TTC3 between STS tissues and normal tissues in Oncomine database.

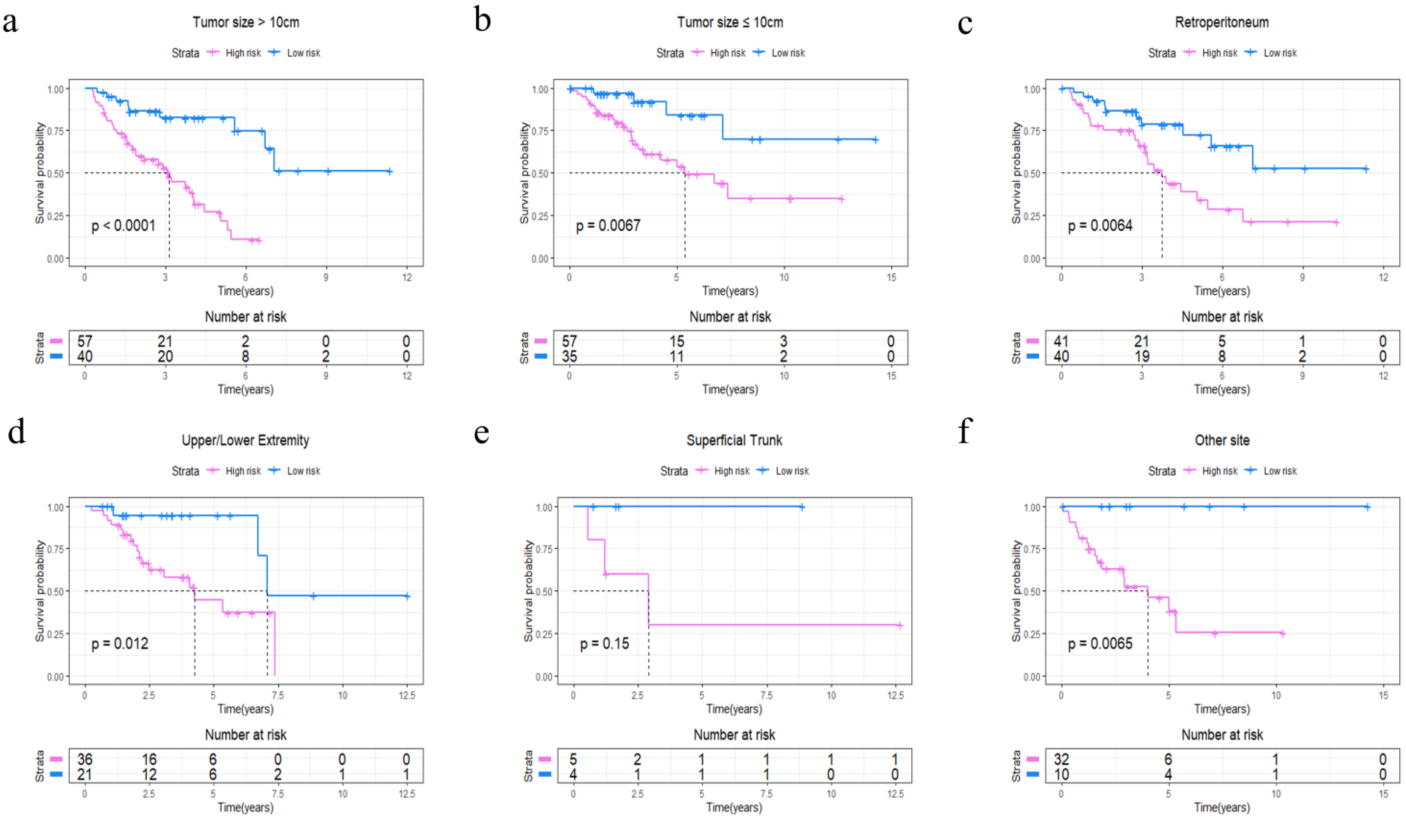

Supplementary Figure S3. OS analysis of different tumor size groups and tumor site groups based on the risk model in the whole set of TCGA cohort. Based on the risk score model, stratified OS analysis performed in patients with different tumor size (a,b) and tumor site (c-f) in the whole set of TCGA cohort. Significance for survival analysis was calculated using a log-rank test, with the red line representing the high-risk group and the blue line representing the low-risk group. The grouping of STS samples is shown at the bottom of the charts.

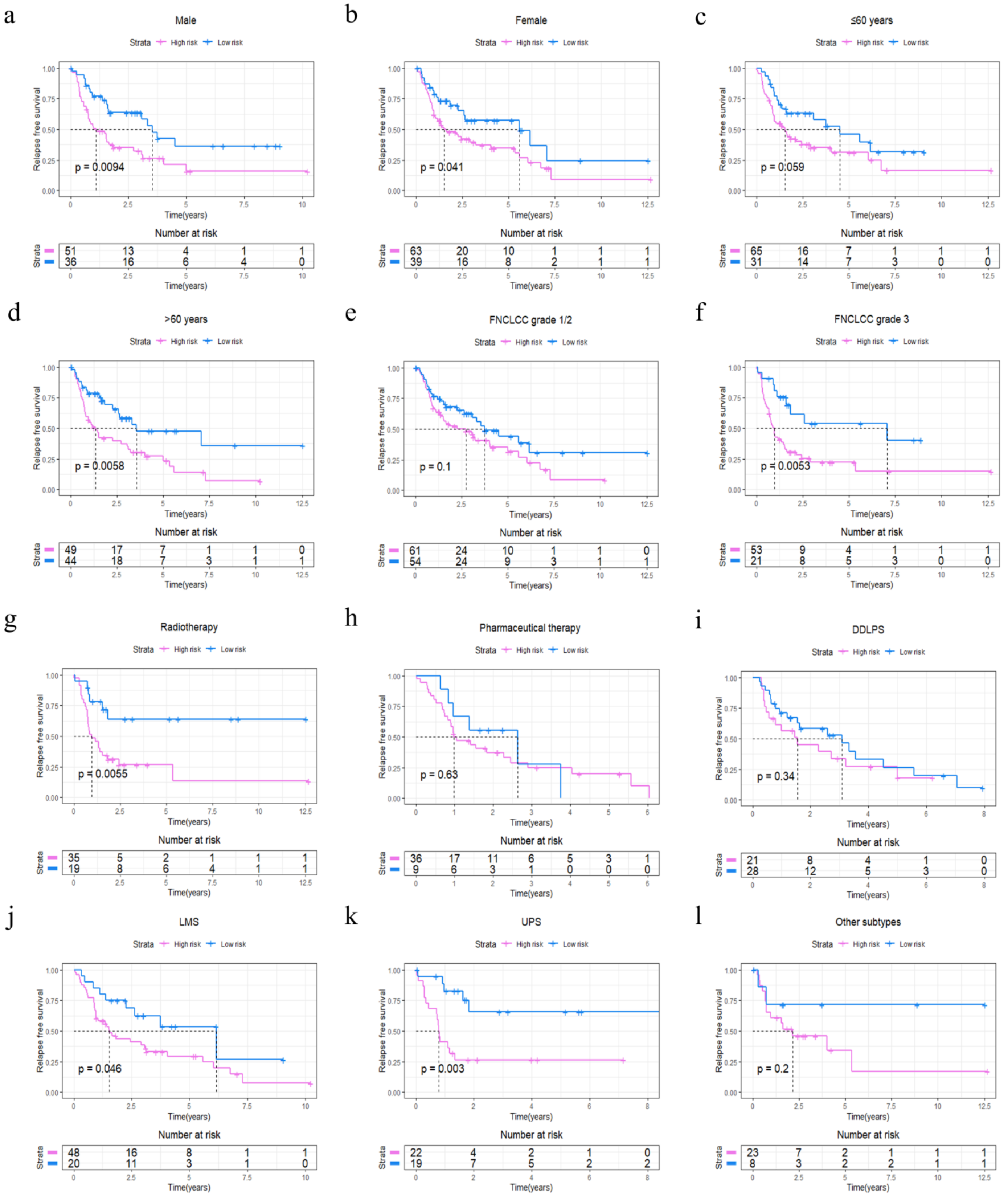

Supplementary Figure S4. Stratified RFS analysis based on the risk model in the whole set of TCGA cohort. Based on the risk score model, stratified RFS analysis performed in patients with different clinical parameters, such as gender (**a** and **b**), age

group (**c** and **d**), FNCLCC grade (**e** and **f**), radiotherapy (**g**), pharmaceutical therapy (**h**) and histological type (**i-l**) in the whole set of TCGA cohort. Significance for survival analysis was calculated using a log-rank test, with the red line representing the high-risk group and the blue line representing the low-risk group. The grouping of STS samples is shown at the bottom of the charts.

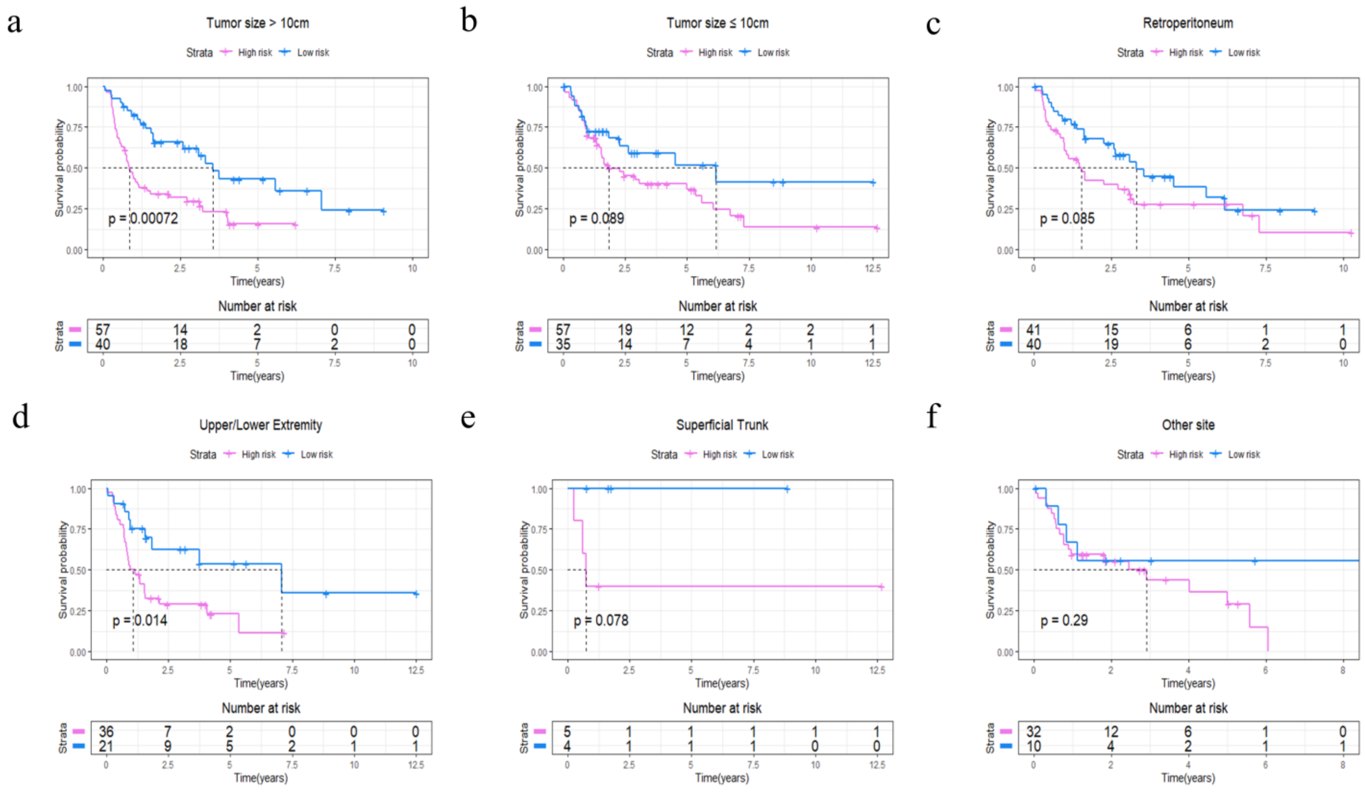

Supplementary Figure S5. RFS analysis of different tumor size groups and tumor site groups based on the risk model in the whole set of TCGA cohort. Based on the risk score model, stratified RFS analysis performed in patients with different tumor size (a,b) and tumor site (c-f) in the whole set of TCGA cohort.. Significance for survival analysis was calculated using a log-rank test, with the red line representing the high-risk group and the blue line representing the low-risk group. The grouping of STS samples is shown at the bottom of the charts.

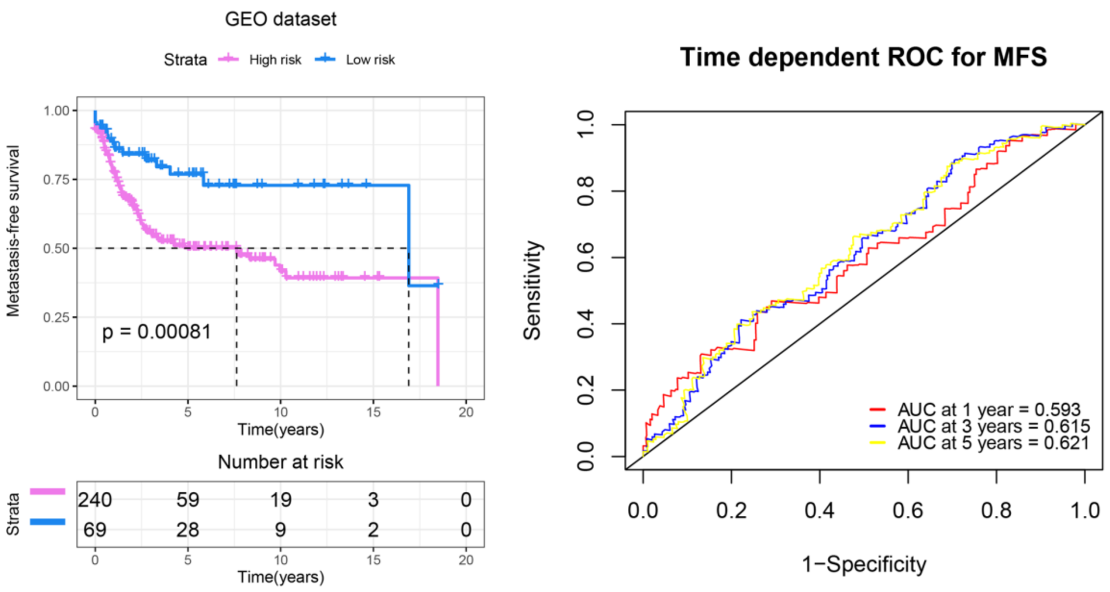

Supplementary Figure S6. Kaplan–Meier analysis and time-dependent ROC analysis of MFS for the four-gene signature in STS.

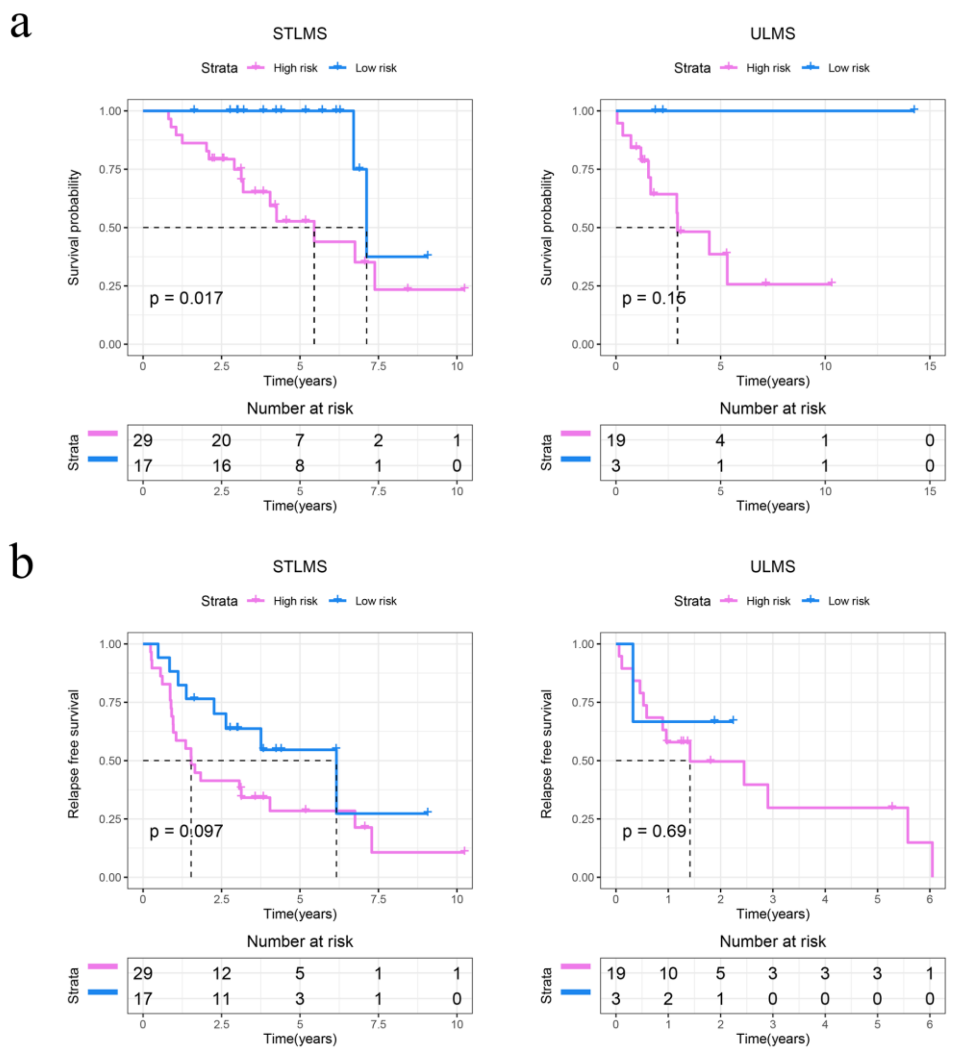

Supplementary Figure S7. Kaplan–Meier analysis of OS (a) and RFS (b) for the four-gene signature in STLMS and ULMS.

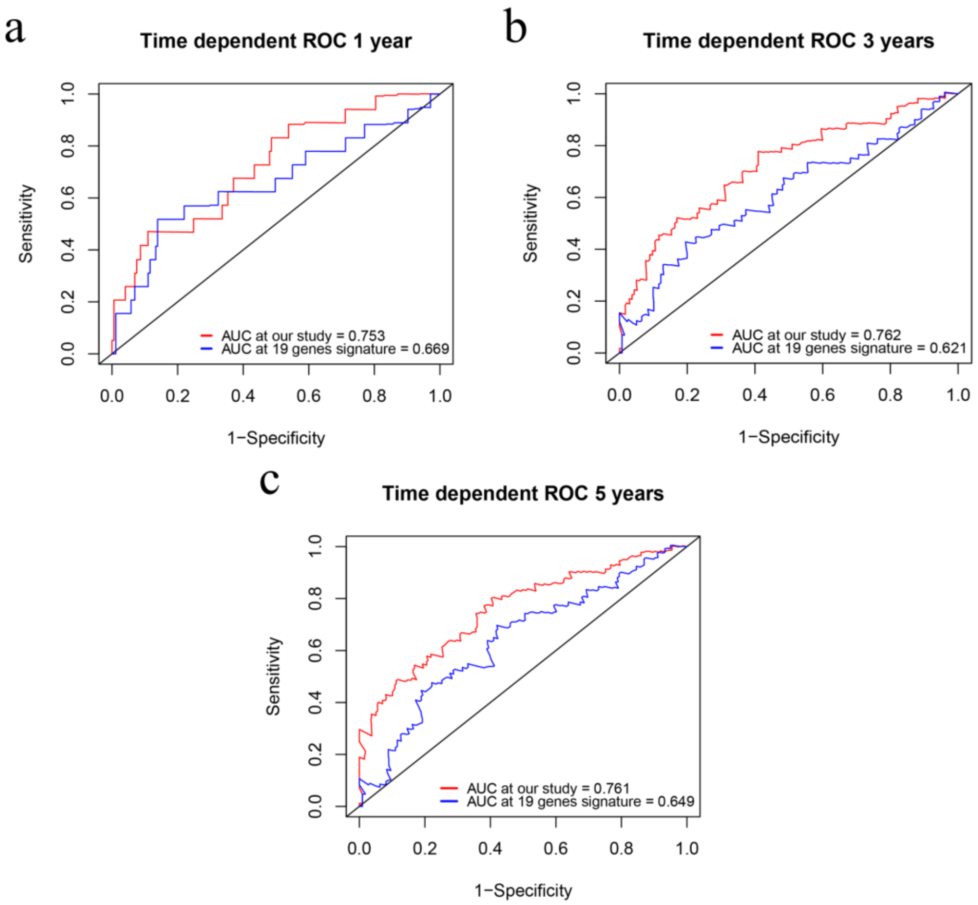

Supplementary Figure S8. Comparison of our four-genes signature and 19 genes signature of Shen et al.
